# Divergent gut microbial metabolism supports niche partitioning in giant and red pandas

**DOI:** 10.3389/fmicb.2025.1698108

**Published:** 2025-11-25

**Authors:** Yanshan Zhou, Dunwu Qi, Chao Chen, Wenlei Bi, Xiang Yu, Jiabin Liu, Guanwei Lan, Rong Hou, Zusheng Li, Rui Ma

**Affiliations:** The Conservation of Endangered Wildlife Key Laboratory of Sichuan Province, Chengdu Research Base of Giant Panda Breeding, Chengdu, Sichuan, China

**Keywords:** giant pandas, red pandas, gut microbiota, metagenome, niche differentiation

## Abstract

**Introduction:**

The gut microbiota plays a pivotal role in regulating the host's physiological functions and behavior. The coevolutionary relationship between the host and its gut microbiota facilitates adaptation to specific ecological niches. As obligate bamboo feeders, giant pandas (Ailuropoda melanoleuca) and red pandas (Ailurus styani) exhibit distinct feeding preferences: the former primarily consumes bamboo stems and leaves, while the latter feeds mainly on bamboo leaves. This study aims to elucidate how these species adapt metabolically to different parts of bamboo via gut microbial activity.

**Methods:**

We employed 16S rRNA gene sequencing to analyze the structure and function of fecal microbial communities in giant pandas (GP) and red pandas (RP).

**Results:**

Significant differences in gut microbiota composition were observed between the GP and RP groups. Eight core bacterial taxa constituted over 99.97% of the total microbial composition, with the RP group exhibiting higher species richness but lower overall diversity. At the phylum level, Proteobacteria, Bacteroidetes, Actinobacteria, Acidobacteria, and Flavobacteria were significantly enriched in the GP group, whereas Firmicutes dominated in the RP group. At the genus level, Sphingomonas, Methylobacterium, Cryomonas, and Terriglobus were more abundant in the GP group, while Streptococcus and Rhizobium were enriched in the RP group. Functional metabolic analysis indicated that lipid and amino acid metabolism pathways were significantly enriched in the GP group, whereas nucleotide and carbohydrate metabolism pathways were prominent in the RP group. Further analysis revealed that Sphingomonas and Methylobacterium in the GP group positively regulated amino acid and lipid metabolism, while Streptococcus in the RP group enhanced nucleotide and carbohydrate metabolism.

**Discussion:**

These findings suggest that the distinct metabolic pathways of the gut microbiota in giant and red pandas have evolved in concert with their dietary strategies, energy acquisition modes, and ecological niche differentiation, forming a highly coordinated adaptive system.

## 1 Introduction

Convergent evolution of diet represents a key evolutionary paradigm in which animals independently develop parallel adaptive traits to exploit similar ecological niches (Moeller et al., 2013; Delsuc et al., 2014). In the specialized bamboo forest ecosystem, the giant pandas (*Ailuropoda melanoleuca*) and red pandas (*Ailurus styani*) exemplify a unique dual-species model of convergent evolution (Li et al., 2015; Huang et al., 2021). Despite their significant phylogenetic divergence—the giant panda belongs to the *Ursidae*, whereas the red panda is classified under the *Ailuridae* (Wei et al., 2015; Zhao et al., 2024)—both species have independently evolved highly specialized bamboo-based diets (Williams et al., 2014; Mckenney et al., 2017). Notably, the dietary shifts during evolution, along with species-specific preferences for distinct bamboo components, indicate underlying ecological drivers and associated adaptive evolutionary mechanisms (Dhami et al., 2021; Zhang et al., 2024). This case of convergent evolution across divergent families within the order *Carnivora* offers a compelling natural experimental system to investigate how diet shapes the co-adaptive evolution of host organisms and their gut microbiota (Sayak et al., 2015).

As the primary food source for giant pandas and red pandas, mature bamboo contains 40-60% cellulose (dry weight) and 15-25% lignin, with secondary metabolites such as cyanogenic glycosides and phenolic compounds present at significantly higher concentrations compared to other grasses (Xue et al., 2015; Nie et al., 2019). This poses a substantial challenge for giant pandas and red pandas, which retain carnivorous gut characteristics, including a digestive tract length only one-third that of similarly sized herbivores (Wei et al., 1999; Han et al., 2019). Current theoretical models suggest that the obligate bamboo diets of giant pandas and red pandas are sustained through the co-adaptive evolution of their gut microbiota, which enables efficient nutritional metabolism. Key mechanisms include: (1) secretion of cellulase systems to degrade structural polysaccharides (Williams et al., 2014); (2) nitrogen metabolism to compensate for protein deficiencies (Deng et al., 2023; Lu et al., 2024); and (3) detoxification processes, such as the conversion of cyanogenic glycosides into thiocyanate (Deng et al., 2023; Huang et al., 2023; Ning et al., 2024; Zhao et al., 2024). Notably, giant pandas consume 4-6 kg of bamboo fiber daily, yet their apparent digestibility remains below 17%, underscoring the critical role of microbial metabolic compensation (Zhu et al., 2011). Despite this, current research predominantly focuses on describing the microbiota composition of single species, leaving a gap in systematic analyses of functional differentiation mechanisms among sympatrically distributed and convergently feeding species.

It is worth noting that although both giant pandas and red pandas are bamboo-dependent, they exhibit significant dietary niche partitioning (Shan et al., 2018). Giant pandas primarily consume highly lignified bamboo stems and branches rich in structural fiber (Li et al., 2017; Zhang et al., 2018; Yan et al., 2024), whereas red pandas preferentially feed on bamboo leaves that are higher in protein and soluble sugars but lower in fiber content (Kong et al., 2014). This marked divergence in dietary substrates imposes strong natural selection pressures, likely driving adaptive differentiation in the functional profiles and metabolic pathways of their gut microbiota. Recent metagenomic studies reveal that the intestinal microbiota of both species are predominantly composed of Firmicutes and Proteobacteria. The CAZy enzyme system (carbohydrate-active enzymes) encoded in the giant panda's genome partially compensates for the host's insufficient endogenous digestive enzymes (Danlin et al., 2017; Wei et al., 2015; Yao et al., 2019). Meanwhile, the δ15N values in the feces of red pandas are significantly higher than those of giant pandas, suggesting that the red panda's microbiota may possess a more efficient nitrogen cycling pathway (Li et al., 2015). Analyzing the nutritional gradients of bamboo components, bamboo leaves are characterized by high levels of soluble sugars (8-12% dry weight) and crude protein (15-18% dry weight), with relatively low fiber content (NDF 50-55%) (Wang et al., 2017). In contrast, bamboo stems exhibit significantly elevated fiber content (NDF 75-80%), sharply reduced soluble nutrients, and cyanogenic glycoside concentrations that are 2-3 times higher than those in bamboo leaves (Wang et al., 2017). These differences likely drive the functional differentiation of gut microbiota through natural selection pressures (Zhao et al., 2024). Based on these observations, we hypothesize that the gut microbiota of giant pandas have undergone adaptive evolution to enhance fiber degradation and detoxification functions in response to their bamboo stem-based diet, whereas those of red pandas may be more specialized for the rapid metabolism of readily available carbohydrates found in bamboo leaves (Lei et al., 2021; Zhao et al., 2024). Unfortunately, the majority of existing studies have focused on single species and have not adequately controlled for key confounding variables such as diet composition and environmental conditions—factors that are well established to significantly affect gut microbiota composition and functional profiles (Huang et al., 2024). There remains a lack of systematic investigation into how the gut microbiomes of giant pandas and red pandas undergo functional differentiation to accommodate their distinct feeding strategies under standardized conditions, including identical diets and shared captive environments.

This study aims to elucidate how gut microbial functions adaptively respond to host dietary niche differentiation. Using 16S rRNA gene full length sequencing and metagenomic function prediction, we systematically characterize the structural and functional divergence of the gut microbiota in giant pandas and red pandas under strictly controlled dietary and environmental conditions. Specifically, we address two key questions: (1) How are microbial functional modules—particularly those involved in carbohydrate, amino acid, and lipid metabolism—specifically adapted to the distinct dietary substrates preferred by each host (bamboo stems and bamboo leaves)? (2) Do these divergent metabolic pathways constitute a microbial adaptive foundation underlying host ecological niche partitioning? Our findings will refine the theoretical framework of host-microbe coevolution, offer novel insights into convergent evolution and niche differentiation through the lens of microbial functional adaptation, and provide meaningful implications for the conservation biology of endangered species.

## 2 Materials and methods

### 2.1 Research location and subjects

This study was conducted at the Chengdu Research Base of Giant Panda Breeding in Sichuan Province, China, which is a major institution for the *ex-situ* conservation of giant pandas and red pandas. All the research subjects lived in the captive environment of the Chengdu Research Base of Giant Panda Breeding. This study involved 5 adult giant pandas (2 males and 3 females; age = 12.20 ± 1.36) and 6 adult red pandas (3 males and 3 females; age = 6.67 ± 1.33, Supplementary Table 1). The health of all individuals was confirmed to be good throughout the study period by the veterinarians of the Chengdu Research Base of Giant Panda Breeding.

#### 2.1.1 Diet and feeding

To ensure uniform dietary conditions across all study samples, the daily management and feeding protocols for the research subjects were strictly standardized. The primary component of their diet consisted exclusively of fresh *Bashania fargesii*. Throughout the sampling period, this species was the sole bamboo type provided, thereby eliminating potential confounding effects of bamboo species variation on intestinal microbiota composition. In addition to ad libitum access to *Bashania fargesii*, all individuals received identical amounts of a standardized concentrate feed with a consistent formulation (Wang et al., 2017), along with continuous access to clean drinking water. This controlled feeding regimen ensured high consistency in diet type, major components, and nutrient sources across all subjects, establishing a reliable basis for comparing gut microbiota profiles between the two panda groups under identical bamboo-based diets. Importantly, none of the individuals had been administered antibiotics, hormones, or anthelmintic treatments during the sampling period or in the preceding 6 months.

#### 2.1.2 Age and gut microbiota

Under captive conditions, giant pandas can live for over 20 years, whereas red pandas typically have a lifespan of 8 to 14 years. The giant panda and red panda cohorts included in this study are both representative of the middle-life stage within their respective species. Extensive research has established that host age is a key determinant in shaping the composition and functional profile of the gut microbiota (Li et al., 2023). Age-related systemic shifts in microbial composition and metabolic activity may interact with or confound diet-induced variations (Li et al., 2023). However, because both study groups were at comparable life stages relative to their species-specific lifespans, the observed inter-species differences in gut microbiota are more likely attributable to inherent species-specific and dietary factors, rather than disparities in age structure.

### 2.2 Sample collection and grouped

In this study, all samples were collected in February 2024, with sampling consistently conducted between 08:00 and 10:00 AM, corresponding to the peak defecation period for both giant pandas and red pandas. Collection personnel strictly adhered to the sample collection protocols, wearing disposable surgical masks and polyethylene (PE) gloves. Fresh feces were utilized for gut bacterial surveys. Once defecation occurred, the feces were carefully transferred to disposable sterile surgical drapes. The outer layer of the feces was gently removed, and the inner portion was placed into 50 mL sterile cryogenic vials. After proper labeling, the samples were rapidly frozen using sampling transport boxes containing dry ice, ensuring that the gut microbiota remained unaffected by external environmental factors. Subsequently, the samples were transported to the laboratory and stored in a −80 °C ultra-low temperature freezer until DNA extraction could be performed. We categorized the samples into two groups based on the species of individuals: GP group (Giant pandas) and RP group (Red pandas).

### 2.3 Bacterial surveys

#### 2.3.1 Extraction of DNA from fecal samples

The collected fecal samples were pre-treated using the silica bead-based method (Xue et al., 2015). Briefly, each sample was vortexed with Pre-wash Buffer (containing Tris-HCl, EDTA, etc.) at a 10:1 ratio (1 ml buffer per 100 mg feces) to remove contaminants such as bilirubin, bile salts, and plant polysaccharides. Following vortexing, samples were centrifuged at 12,000 rpm for 5 min; the supernatant was discarded and the pellet retained. The pellet was resuspended in 5 ml of cold physiological saline (4 °C), and zirconia/silica beads (0.9-1.1 mm diameter) were added to a final concentration of 0.05 g/L. Mechanical lysis was performed using a high-speed shaking instrument (Analytik Jena AG SpeedMill PLUS) at maximum speed for 10 min to disrupt bacterial cell walls and maximize DNA release, thereby enhancing DNA yield. Total microbial DNA was then extracted using the Qiagen QIAamp DNA Stool Mini Kit (Qiagen, Germany), following the manufacturer's protocol for the lysis step, with minor modifications based on published methods to optimize lysis temperature and improve DNA recovery (Zheng et al., 2014). To prevent cross-contamination from exogenous DNA, all extractions were performed in a single batch under identical conditions. A negative control (extraction blank control) was included by processing all reagents and steps in parallel without adding any fecal material.

DNA quality was assessed via 1% agarose gel electrophoresis. For each sample, the 16S rRNA gene was amplified using the primer pair 27F (5′-AGAGTTTGATCCTGGCTCAG-3′) and 1492R (5′-TACCTTGTTACGACTT-3′) under the following PCR conditions: initial denaturation at 95 °C for 3 min; 30 cycles of amplification (95 °C for 30 s, 56 °C for 30 s, 72 °C for 3 min); final extension at 72 °C for 10 min; and holding at 4 °C. Amplifications were performed on a T100 Thermal Cycler (Bio-Rad Laboratories, USA). To minimize stochastic amplification bias, triplicate PCR reactions were conducted for each sample. Following electrophoretic verification, PCR products were purified using AMPure^®^ PB magnetic beads (Pacific Biosciences, USA) and quantified with a Qubit 4.0 Fluorometer (Thermo Fisher Scientific, USA). Purified amplicons were then shipped on dry ice to Lianchuan Bio-Technology Co., Ltd. (Hangzhou, China) for library construction and data processing.

#### 2.3.2 Library construction and data processing

Purified amplicons were pooled in equimolar ratios and used for DNA library construction with the SMRTbell Express Template Prep Kit 3.0 (Pacific Biosciences, USA). Given the high microbial biomass inherent in fecal samples and the uniform processing of all samples under identical library preparation and sequencing conditions-thereby minimizing technical batch effects-a negative control library was not included in this step (Liu et al., 2025; Wu et al., 2025). Sequencing was carried out on a PacBio Sequel IIe system at Lianchuan Bio-Technology Co., Ltd. (Hangzhou, China). Circular consensus sequencing (CCS) was performed using SMRT Link v11.0 to generate high-fidelity (HiFi) reads from subreads. For sequence preprocessing and denoising, raw HiFi reads were first demultiplexed by barcode and subjected to length filtering to exclude sequences shorter than 1,000 bp or longer than 1,800 bp. Denoising was conducted using the DADA2 plugin (version 2) within QIIME2 (version 2020.2) (Callahan et al., 2016; Hall and Beiko, 2018) to infer exact amplicon sequence variants (ASVs). Key parameters were configured as follows: due to the inherently high accuracy of PacBio HiFi circular consensus reads, quality-based read truncation was disabled (–*p*-trunc-len 0); the maximum expected errors per read (–*p*-max-ee) was set to 20.0; chimeras were removed using the consensus method (–*p*-chimera-method consensus) with a minimum parent fold abundance threshold of 3.5 (–*p*-min-fold-parent-over-abundance); and analyses were executed in parallel across 20 computational threads (–*p*-n-threads 20). Following denoising, an ASV table was generated, from which chloroplast and mitochondrial sequences were subsequently removed.

Rarefaction and taxonomic analysis. To minimize the impact of sequencing depth on downstream alpha and beta diversity analyses, the ASV table was rarefied to a threshold of 11,000 sequences per sample—the maximum depth at which all 11 samples were retained without data loss. Detailed sequencing metrics for each sample, including read counts before and after rarefaction, were provided in Supplementary Table 2. The rarefaction curve plateaued at this depth, indicating that the sequencing effort was sufficient to capture the majority of microbial diversity present (Supplementary Figure 1). Average sequence coverage across all samples, as estimated by Good's coverage, exceeded 99%. Taxonomic assignment of ASVs was performed using a Naive Bayes classifier implemented in QIIME2, trained against the SILVA 16S rRNA gene database (version 138).

### 2.4 Statistical analysis

Taxonomic annotation of the obtained ASVs was performed using QIIME2 (version 2020.2), and downstream analyses were carried out via the Lianchuan Bio OmicStudio Cloud Platform (https://www.omicstudio.cn) (Lyu et al., 2023). These analyses included the identification of taxa with a relative abundance exceeding 0.1% in each sample or group across multiple taxonomic levels—phylum, class, order, family, genus, and species—using R (4.5.0) and the ggplot2 package (3.5.2), with feature selection guided by maximum abundance ranking. Bar plots summarizing taxonomic composition were generated using ggplot2 (3.5.2), while Venn diagrams were constructed using the VennDiagram package (1.7.3) to visualize shared and unique ASVs among groups. Alpha diversity indices—including ACE, Chao1, Shannon, and Simpson—were calculated using the vegan package (2.7.1) in R (4.5.0). Group-wise comparisons of alpha diversity were conducted using the two-sided Wilcoxon rank-sum test, and *p*-values were adjusted for multiple testing using the Benjamini-Hochberg procedure to control the false discovery rate (FDR).

Principal coordinate analysis (PCoA) was performed based on ASV composition using the Bray-Curtis dissimilarity metric and the vegan package (2.7.1) in R (4.5.0) to assess similarities and differences in microbial community structure across samples. To statistically evaluate pairwise group differences in beta diversity, permutational multivariate analysis of variance (Adonis) and analysis of similarities (ANOSIM) were conducted, both with 999 permutations to determine statistical significance. Relative abundances of the top seven phyla and top ten genera in the GP and RP groups are visualized as bar plots in GraphPad Prism 9.

To identify microbial taxa that significant differences between groups, a multi-level differential abundance analysis was performed using the LEfSe (Linear Discriminant Analysis Effect Size) software (version 1.1.2), via its official Galaxy platform (https://huttenhower.sph.harvard.edu/lefse). The LEfSe pipeline comprises three sequential steps: first, a non-parametric Kruskal-Wallis rank-sum test (*p* < 0.05) was used to detect features exhibiting significant abundance differences across groups; second, pairwise Wilcoxon rank-sum tests (*p* < 0.05) were applied to confirm consistent directional enrichment within specific groups; and third, linear discriminant analysis (LDA) was employed to estimate the effect size of each differentially abundant feature. Taxa with an LDA score >4.0 were considered biologically meaningful discriminators. The resulting differentially enriched phyla and genera meeting this threshold were visualized in GraphPad Prism 9.

The functional potential of the microbial community was predicted using PICRUSt2 (2.2.0) (Wang et al., 2007), based on the KEGG database (release 93.1). The analysis was performed using default parameters without any modifications to ensure reproducibility according to the standard pipeline. The ASV sequences were mapped to the reference database to infer their genomic content, followed by the prediction of gene families and the calculation of relative abundances for KEGG Orthologs (KOs) and metabolic pathways across KEGG levels 1, 2, and 3. The complete analysis script is publicly available in the official PICRUSt2 GitHub repository (https://github.com/picrust/picrust2) and was executed as per the default workflow. Differential metabolic pathways at KEGG level 2 between the two groups were identified using the two-sided Wilcoxon rank-sum test, with *p*-values adjusted for multiple comparisons using the Benjamini-Hochberg false discovery rate (FDR) correction. A heatmap visualizing these differences was generated using GraphPad Prism 9. To assess associations between differentially abundant genera and metabolic pathways, Spearman's rank correlation coefficient was computed, with statistical significance set at *p* < 0.05. For genus-pathway pairs showing significant correlations, univariate linear regression analysis was further conducted in GraphPad Prism 9 to validate the strength and direction of these relationships. To account for multiple testing across those different genus-pathway pairs, the resulting *p*-values were adjusted using the Benjamini-Hochberg FDR correction method to generate q-values. Statistical significance for the correlation analysis in the univariate linear regression was determined using a threshold of *q* < 0.05. To determine an appropriate coefficient of determination (*R*^2^) threshold for identifying robust genus-pathway relationships, A systematic sensitivity analysis was performed to evaluating multiple *R*^2^ cut-offs (Zuñiga et al., 2017). We evaluated the outcomes across a gradient of *R*^2^ thresholds (*R*^2^ < 0.3, 0.3 ≤ *R*^2^ < 0.4, 0.4 ≤ *R*^2^ < 0.5, and *R*^2^ ≥ 0.5) in conjunction with the significance threshold (*q* < 0.05). This analysis aimed to quantify the trade-off between the stringency of the effect size filter (controlling false positives) and statistical power (minimizing false negatives). The results of this evaluation are presented as a heatmap in GraphPad Prism 9, and a network illustrating robust genus-metabolic pathway correlations was constructed and visualized using the igraph package (2.0.3) in R (4.5.0). In this study, statistical significance was defined as *p* < 0.05, with highly significant differences designated at *p* < 0.01 and *p* < 0.001.

### 2.5 Ethical approval

The research complied with methods and experimental protocols approved by the Institutional Animal Care and Use Committee and conformed to Chengdu Research Base of Giant Panda Breeding, Sichuan Province, China (IACUC No. 2024016). This study is performed in accordance with relevant guidelines and regulations. All methods are reported in accordance with ARRIVE guidelines (https://arriveguidelines.org).

## 3 Results

### 3.1 Sequencing information

After the raw data was filtered and spliced, a total of 133,338 high-quality clean reads were produced in the GP and RP groups, with an average of 12,121 sequences per sample (ranging from 11,124 to 13,430, Supplementary Table 2, Supplementary Figure 2). The average sequence length was 1,481 bp, with the maximum length being 1,461 bp and the shortest length being 1,443 bp (Supplementary Table 2, Supplementary Figure 2).

### 3.2 Gut microbial diversity and community analysis

**Figure 1:**
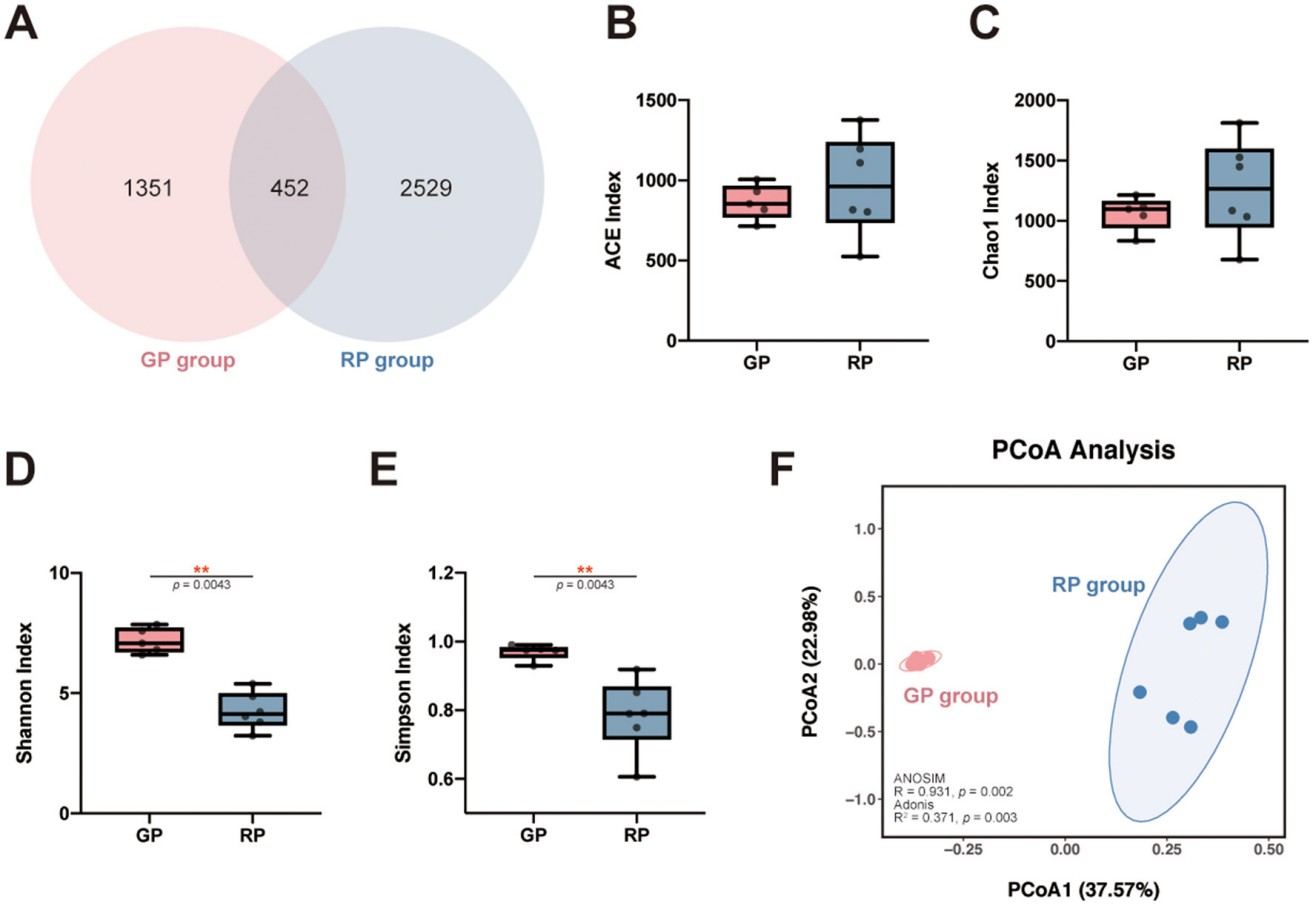
ASV distribution and microbial diversity across groups. **(A)** Venn diagram showing shared and unique ASVs among the GP and RP groups. **(B-E)** The gut microbiota comparisons: ACE **(B)** and Chao1 **(C)** richness indices, and Shannon **(D)** and Simpson **(E)** diversity indices, presented as boxplots with individual samples displayed as dots. **(F)** PCoA plot based on Bray-Curtis dissimilarity, illustrating intergroup differences in microbial community structure between the GP group (red) and RP group (blue). The first two principal coordinates explain 37.57% and 22.98% of the total variation, respectively. * indicates significant differences (0.01 < adjusted *p*-value (FDR) < 0.05); ** indicates highly significant differences (0.001 < adjusted *p*-value (FDR) < 0.01).

The total numbers of ASVs obtained was 4,332, among which 452 ASVs were shared by both groups, 1,351 and 2,529 ASVs were uniquely identified in the GP and the RP groups, respectively (Figure 1A). The ACE (*p*_*FDR*_ = 0.792) and Chao1 (*p*_*FDR*_ = 0.662) richness indices were higher in the RP group compared to the GP group, as determined by the Wilcoxon rank-sum test, although these differences were not statistically significant (Figures 1B, C, Supplementary Table 3). In contrast, the Shannon (*p*_*FDR*_ = 0.004) and Simpson (*p*_*FDR*_ = 0.004) diversity indices were significantly higher in the GP group than in the RP group (Figures 1D, E, Supplementary Table 3). Our results indicate that the gut microbiota in the RP group exhibits higher species richness but lower diversity. Diversity analysis reveals greater dispersion of microbial samples in the RP group, with a clear separation from the GP group in the PCoA plot, where the first two principal coordinates explain 37.57% and 22.98% of the total variation, respectively (Figure 1F). This structural distinction is further supported by ANOSIM (*R* = 0.931, *p* = 0.002) and Adonis (*R*^2^ = 0.371, *p* = 0.004), demonstrating significant differences in the compositional structure of the gut microbial communities between the RP and GP groups.

### 3.3 Gut microbial composition analysis

**Figure 2:**
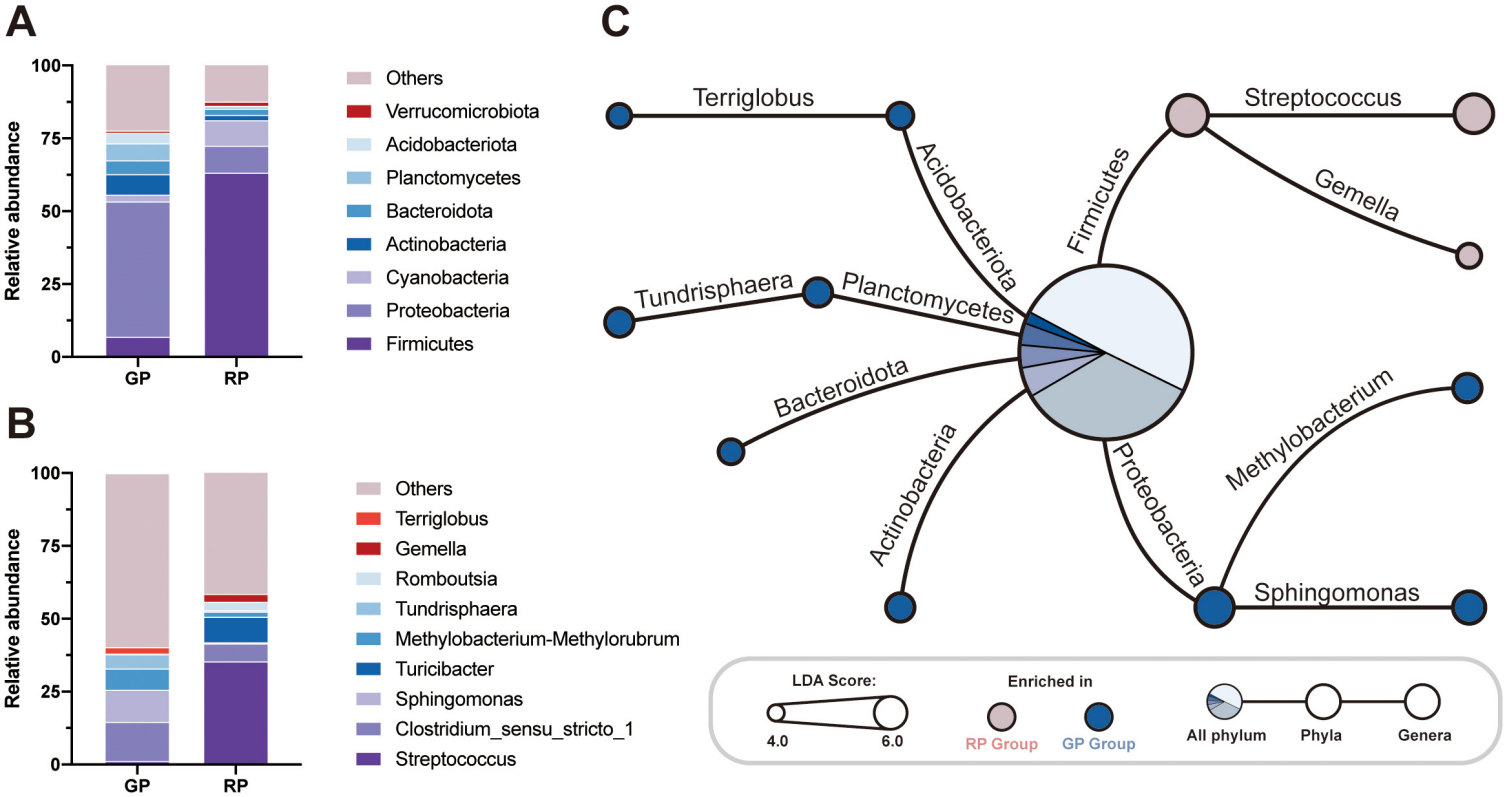
The gut microbiota and metabolism pathways surveys. The relative abundance analysis of **(A)** all the phyla and **(B)** the top 10 genera in WS and SA. **(C)** The Linear Discriminant Analysis (LDA) demonstrated distinct microorganism on phylum level and genus level enriched in the GP and RP groups. When the default LDA value is > 4.0 and the *p* value is < 0.05, the result corresponds to a differential species; The red dots red represent a phylum or genus enriched in RP group, The blue dots red represent a phylum or genus enriched in GP group; The central pie chart represents the relative abundance of significantly different phyla as a percentage of all differential phyla.

The bacterial distribution was characterized in terms of the relative taxonomic abundances. A total of 80 phyla, 127 classes, 219 orders, 339 families, 739 genera, 668 species and 4,332 ASVs were detected in the fecal samples. On the phylum level, the GP and RP groups shared the same eight core bacterial species (relative abundance >1%), and the top eight relative abundances of phyla, ranked from highest to lowest, were Firmicutes (GP = 6.98%, RP = 63.20%), Proteobacteria (GP = 46.37%, RP = 9.26%), Cyanobacteria (GP = 2.29%, RP = 8.74%), Actinobacteria (WS = 46.37%, SA = 9.26%), Bacteroidota (WS = 46.37%, SA = 9.26%), Planctomycetes (WS = 46.37%, SA = 9.26%), Acidobacteriota (WS = 46.37%, SA = 9.26%) and Verrucomicrobiota (WS = 46.37%, SA = 9.26%), collectively accounting for more than 99.97% of the total microbial phyla (Figure 2A). The dominant bacterial genera (relative abundance >1%) in the GP group was *Clostridium sensu stricto 1* (13.44%), followed by *Sphingomonas* (10.96%), *Methylobacterium* (7.29%), *Tundrisphaera* (4.90%), *Terriglobus*, (2.27%) and *Streptococcus* (1.20%, Figure 2A). The dominant bacterial genera (relative abundance >1%) in the RP group was *Streptococcus* (35.39%), followed by *Turicibacter* (8.79%), *Clostridium sensu stricto 1* (6.14%), *Romboutsia* (2.89%), *Gemella*, (2.71%), and *Methylobacterium* (1.83%, Figure 2B).

By LDA analysis, we found that the relative abundance of Firmicutes in GP group was significantly lower than that in RP group (LDA Score = 5.451, *p* = 0.006), while the phyla Proteobacteria (LDA Score = 5.262, *p* = 0.006), Planctomycetes (LDA Score = 4.401, *p* = 0.006), Actinobacteria (LDA Score = 4.401, *p* = 0.018), Acidobacteriota (LDA Score = 4.240, *p* = 0.006) and Bacteroidota (LDA Score = 4.117, *p* = 0.018) was significantly enriched in GP group (Figure 2C, Supplementary Table 4). Similarity, we found that the relative abundance of *Streptococcus* (LDA Score = 5.280, *p* = 0.006) and *Gemella* (LDA Score = 4.042, *p* = 0.006) in GP group was significantly lower than those in RP, while the genera *Sphingomonas* (LDA Score = 4.732, *p* = 0.006), *Methylobacterium* (LDA Score = 4.404, *p* = 0.006), *Tundrisphaera* (LDA Score = 4.351, *p* = 0.006) and *Terriglobus* (LDA Score = 4.055, *p* = 0.006) were significantly enriched in GP group (Figure 2C, Supplementary Table 4).

### 3.4 Functional metabolic pathways and correlation analysis of microbial communities

By comparing with the KEGG database, a total of 41 KEGG level 2 metabolic pathways were annotated across the samples from all two groups. The top 5 pathways in relative abundance accounted for 47% of the total pathways, which were: membrane transport (GP = 10.65%, RP= 13.87%), carbohydrate metabolism (GP = 9.90%, RP = 10.56%), amino acid metabolism (GP = 10.24%, RP = 9.05%), energy Metabolism (GP = 6.91%, RP = 6.41%), and replication and repair (GP = 6.87%, RP = 8.20%).

**Figure 3:**
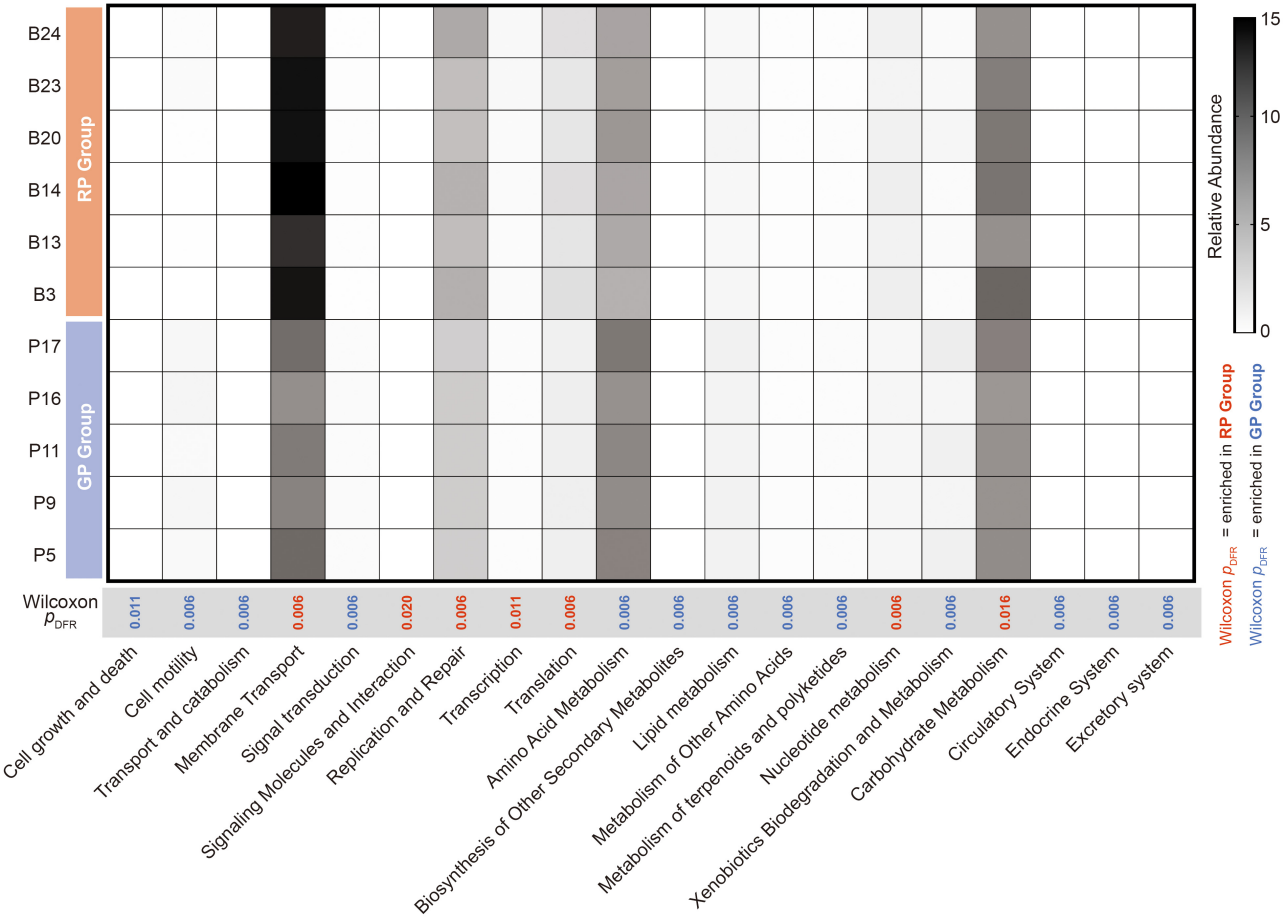
Differential analysis of KEGG level 2 metabolic pathways between the GP and RP groups. Red bars represent pathways significantly enriched in the RP group (Wilcoxon rank-sum test, *p*_*FDR*_ < 0.05), while blue bars indicate pathways enriched in the GP group (*p*_*FDR*_ < 0.05).

The Wilcoxon rank-sum test with FDR correction identified 20 significantly different metabolic pathways between GP and RP groups (Figure 3). Among them, 13 metabolic pathways were enriched in GP group and last 7 metabolic pathways were enriched in RP group. The relative abundance of the membrane transport (*p*_*FDR*_ = 0.006), signaling molecules and Interaction (*p*_*FDR*_ = 0.020), replication and repair (*p*_*FDR*_ = 0.006), transcription (*p*_*FDR*_ = 0.011), translation (*p*_*FDR*_ = 0.006), nucleotide metabolism (*p*_*FDR*_ = 0.006) and carbohydrate metabolism (*p*_*FDR*_ = 0.016) were significantly enriched in RP group. The relative abundance of the cell growth and death (*p*_*FDR*_ = 0.011), cell motility, transport and catabolism, signal transduction, amino acid metabolism, biosynthesis of other secondary metabolites, lipid metabolism, metabolism of other amino acids, metabolism of terpenoids and polyketides, xenobiotics biodegradation and metabolism, circulatory system, endocrine system and excretory system were significantly enriched in GP group (*p*_*FDR*_ = 0.006, Figure 3, Supplementary Table 5).

Further Spearman correlation analysis was performed to explore the potential relationships between differential genera and metabolic pathways. The results showed that the relative abundance of the genera *Streptococcus* and *Gemella* were significantly positively correlated with the relative abundance of 2 metabolic pathways in environmental information processing functions, 3 metabolic pathways in genetic information processing functions and 2 metabolic pathways in metabolism functions (*Streptococcus*: nucleotide metabolism, *r* = 0.809, *p* = 0.004; carbohydrate metabolism, *r* = 0.709, *p* = 0.019; *Gemella*: nucleotide metabolism, *r* = 0.817, *p* = 0.002; Supplementary Table 6). The remaining four differential genera enriched in the GP group were significantly associated with functions related to cellular processes, organismal systems, and various metabolism pathways, including amino acid metabolism, biosynthesis of other secondary metabolites, lipid metabolism, metabolism of other amino acids, metabolism of terpenoids and polyketides, and xenobiotics biodegradation and metabolism (Supplementary Table 6).

**Figure 4:**
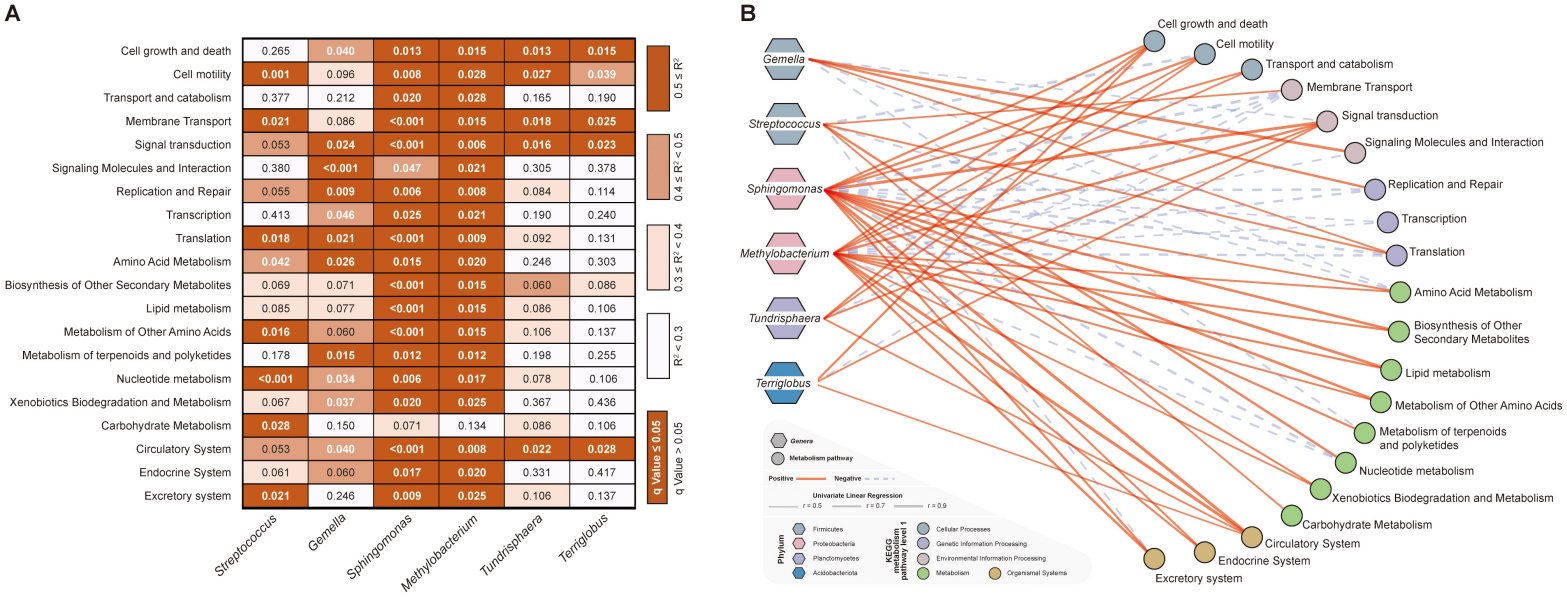
Univariate linear regression sensitivity and network visualization of differential bacterial genera in relation to KEGG level 2 metabolic pathways. **(A)** Heatmap of univariate linear regression *R*^2^ values between differential bacterial genera and KEGG level 2 metabolic pathways. Each column represents a differential bacterial genus, and each row represents a KEGG level 2 pathway. The background color indicates the R^2^ range: white (R^2^ < 0.3), light orchid (0.3 ≤ *R*^2^ < 0.4), orange (0.4 ≤ *R*^2^ < 0.5), and dark orange (*R*^2^ ≥ 0.5). Numerical values denote the q-values from regression analysis; bold white text indicates q < 0.05 (statistically significant), while thin black text indicates q ≥ 0.05. **(B)** Network visualization of significant associations. Hexagons represent differential bacterial genera, circles represent KEGG level 2 metabolic pathways. Red solid lines indicate positive correlations, blue dotted lines indicate negative correlations, with line thickness proportional to the strength of the association.

The systematic sensitivity analysis revealed a clear trade-off between the number of associations identified and their reliability (Figure 4A). Specifically, at the *R*^2^ ≥ 0.5 threshold, we identified 59 robust correlations, with a false-positive rate of only 0% among the selected associations (as all passed *q* < 0.05, Figure 4A). In contrast, lower thresholds captured more associations but introduced a substantially higher proportion of potential false positives (0.3 ≤ *R*^2^ < 0.4, 19.67% of all tested pairs; 0.4 ≤ *R*^2^ < 0.5, 5.00% of all tested pairs, Figure 4A). Therefore, the *R*^2^> 0.5 threshold was selected to ensure the high reliability and robustness of the reported key relationships, which is a prudent strategy given the exploratory nature of the study and the need to generate hypotheses for future validation.

At last, the univariate linear regression analysis was applied to examine the correlations between different bacterial genera and different metabolic pathways (set thresholds: *R*^2^ > 0.5, *p* < 0.05). After excluding redundant information, we identified several key relationships. Specifically, the two genera (*Sphingomonas* and *Methylobacterium*) enriched in the GP group, primarily positively regulated the functions related to cellular processes, organismal systems, as well as the metabolic pathways of amino acid metabolism, biosynthesis of other secondary metabolites, lipid metabolism, metabolism of other amino acids, metabolism of terpenoids and polyketides, xenobiotics biodegradation and metabolism (Figure 4B, Supplementary Table 7). In contrast, the other two genera enriched in the GP group (*Tundrisphaera* and *Terriglobus*) mainly positively regulated the metabolic pathways of cell growth and death, signal transduction, and circulatory system. On the other hand, Genera *Gamella*, enriched in the RP group, primarily positively regulated the metabolic pathways of signaling molecules and interaction (*R*^2^ = 0.844, *q* < 0.001), replication and repair (*R*^2^ = 0.702, *q* < 0.001), and translation (*R*^2^ = 0.567, *q* = 0.021), while Genera *Streptococcus* significantly enhanced the nucleotide metabolism (*R*^2^ = 0.765, *q* < 0.001) and carbohydrate metabolism (*R*^2^= 0.509, *q* = 0.028) pathways in the host gut microbiota (Figure 4B, Supplementary Table 7).

## 4 Discussion

This study compares the gut microbiomes of giant and red pandas under identical environmental conditions, demonstrating that their functional divergence is not due to random interspecies variation, but rather reflects a highly adaptive evolutionary response shaped by their distinct dietary niches. The observed differences in metabolic pathways are closely aligned with host-specific dietary strategies, energy acquisition mechanisms, and ecological niche partitioning. Our findings revealed that the gut microbiota of giant and red pandas have evolved divergent energy metabolism profiles, specifically tailored to the nutritional characteristics of their preferred food sources. The genera *Sphingomonas* and *Methylobacterium*, which are significantly enriched in the gut microbiota of giant pandas, have developed a “slow fermentation–detoxification” strategy through enhanced amino acid and lipid metabolic pathways. This strategy represents a direct adaptation to their high-fiber, low-protein diet (Deng et al., 2023). Specifically, *Sphingomonas* may compensate for the host's limited intake of essential amino acids from bamboo stems (Wei et al., 2018; Ning et al., 2024), while the *Methylobacterium*-mediated β-oxidation system likely enables efficient utilization of trace lipids present in bamboo stems (Allen et al., 2009). Notably, the high lignin-cellulose complex content in bamboo stems (>60%) results in an energy density only 40%-50% that of bamboo leaves (Jia et al., 2007). Therefore, this metabolic strategy may represent a physiological adaptation to the low-energy demands associated with the giant panda's sedentary lifestyle (Huang et al., 2022). In contrast, the “fast glycolysis” strategy dominated by *Streptococcus* in the red panda gut is a highly specialized adaptation to sugar-rich bamboo leaves. Soluble sugars in bamboo leaves constitute non-structural carbohydrates (Kong et al., 2014; Chen et al., 2024), serving as ideal substrates for rapid monosaccharide breakdown and ATP production via the phosphotransferase system. This efficient, immediate energy supply mechanism likely supports the energetically demanding arboreal activities of red pandas (Liu et al., 2011). Furthermore, the upregulation of nucleotide metabolism may reduce host energy expenditure on de novo nucleic acid synthesis through salvage pathways, reflecting an optimization of metabolic efficiency under energy-sufficient conditions (Shen et al., 2024; Xia et al., 2022).

At the detoxification level, the functional differences in the gut microbiota of giant and red pandas reflect distinct adaptations to differential exposure to dietary toxins (Zhu et al., 2018). The significant enrichment of xenobiotic degradation pathways in the giant panda's gut microbiome represents a direct evolutionary response to its long-term consumption of bamboo stems rich in phenolic compounds (Deng et al., 2023). Key microbial detoxification mechanisms may serve as a “microbial barrier”, such as aromatic ring-hydroxylating dioxygenases in *Sphingomonas*, it effectively reducing the hepatic detoxification burden on the host. This constitutes a physiological and biochemical compensation mechanism for coping with a high-toxin diet (Shamjana et al., 2024). In contrast, despite higher cyanogenic glycoside content in their bamboo leaf diet, red pandas lack significantly enriched microbial pathways for cyanide detoxification (Xia et al., 2022). This apparent paradox highlights the diversity of adaptive strategies in nature. Instead, red pandas rely on selective feeding behaviors to minimize toxin intake, forming a complementary defense strategy that integrates behavioral avoidance with microbial adaptation (Liu et al., 2022). When such pre-ingestive behavioral filtering substantially reduces toxin exposure, maintaining energetically costly microbial detoxification systems may not represent an evolutionarily optimal allocation of resources (Zhao et al., 2024). From the perspective of ecological niche differentiation, the divergent microbial functions in the two species exemplify the principles of resource allocation theory at the microbiome level. Giant pandas leverage their microbiota's capacity for detoxification and slow fermentation to exploit fibrous bamboo stems, while red pandas dominate the high-energy bamboo leaf niche through specialized rapid glycolysis. This microbially driven “functional complementarity-resource partitioning” coexistence model bears striking similarity to dietary niche segregation observed between African elephants and other herbivores (Rajbhandari et al., 2025). These findings provide novel microbial ecological evidence explaining the stable coexistence of giant and red pandas in the wild.

Our findings provide significant practical guidance for the health management of captive animal populations, demonstrating that dietary formulations in artificial breeding should align with the functional demands of the gut microbiota. For giant pandas in captivity, it is recommended to increase the proportion of bamboo stems in their diet. This recommendation is based on the unique physicochemical properties of the lignin-cellulose complex in bamboo stems, whose three-dimensional network structure not only serves as a source of slowly released dietary fiber but also provides an exclusive ecological niche for *Sphingomonas* and other lignin-degrading bacteria (Shamjana et al., 2024). When the bamboo stem content exceeds the 55% threshold, the abundance of *Sphingomonas* in the gut increases by approximately 23% (Xia et al., 2022; Shamjana et al., 2024), a result that aligns closely with the metabolic pathway profiles of the giant panda gut microbiome. Notably, this bacterial genus converts phenolic acids from bamboo stems into secondary bile acids, such as deoxycholic acid, via the phenylpropanoid metabolic pathway, playing a key role in maintaining liver detoxification function (Ning et al., 2024). While optimizing nutritional intake, the provision of high-sugar fruits should be strictly limited, as dietary D-glucose can significantly suppress the transcriptional activity of phenolic acid-degrading enzymes through the cAMP-CRP signaling pathway (Wang et al., 2020). For red pandas, freshness of bamboo leaves should be prioritized in nutritional management. Our research demonstrates that the carbohydrate metabolic pathway dominated by *Streptococcus* in the gut of red pandas reflects their metabolic strategy for high-sugar bamboo leaves. To ensure the retention of non-structural carbohydrates, we recommend preserving bamboo leaves using vacuum refrigeration. Studies indicate that vacuum pre-cooling combined with strong light sterilization technology can enhance the retention rate of chlorophyll b in bamboo leaves to up to 96.5% (Ray and Ali, 2017).

This study reveals the patterns of metabolic functional differentiation in the gut microbiota of giant and red pandas at the ecological niche level, a finding of particular relevance to their “reintroduction” programs. It underscores the importance of microbial functional flexibility in species conservation and management. However, we must candidly acknowledge several limitations. First, our assessment of the functional potential of the gut microbiota was based on 16S rRNA gene full length sequencing data and PICRUSt2 inference. All conclusions regarding metabolic functions are therefore derived from bioinformatic predictions rather than direct empirical measurements of gene expression or metabolite profiles. The accuracy of these predictions depends on the completeness and taxonomic coverage of reference databases and may overlook fine-scale, species-level metabolic variations. Future studies employing direct molecular profiling techniques will be essential to validate the predicted adaptive functions. Nonetheless, we maintain that PICRUSt2-based functional predictions provide a reasonable and insightful framework for elucidating the potential ecological roles of gut microbiota in these two species. Second, we acknowledge a key limitation: incomplete control over age structure between giant panda and red panda samples. Host age is a well-established driver of gut microbiota composition (Li et al., 2023), and thus some observed differences may reflect age-related effects rather than purely species-specific adaptations. Additionally, the limited sample size and exclusive reliance on captive individuals constrain the generalizability of our findings to wild populations. Small sample sizes inherently reduce statistical power—a common challenge in endangered species research, particularly in wildlife microbiome studies (Yao et al., 2019). To address these limitations, future research should expand sample sizes to include comprehensive age gradients (juveniles, subadults, adults, and elderly), conduct *in vitro* functional validation of microbiome-host phenotype relationships, explore fecal microbiota transplantation experiments, and perform rigorously age-matched cross-species comparisons to more precisely identify the drivers of microbial functional divergence. Despite its constraints, this study provides critical preliminary evidence and offers actionable scientific guidance for future conservation strategies.

## Data Availability

The Full-length raw sequences of the V1-V9 hypervariable region of the 16S rRNA gene for all samples, in FASTQ file format, are available in the NCBI under BioProject accession number: PRJNA1281840 https://www.ncbi.nlm.nih.gov/bioproject/PRJNA1281840.

## References

[B1] Allen M. S. Bradford B. J. Oba M. (2009). Board invited review: the hepatic oxidation theory of the control of feed intake and its application to ruminants. J. Ani. Sci. 87, 3317–3334. doi: 10.2527/jas.2009-1779 19648500

[B2] Callahan B. J. McMurdie P. J. Rosen M. J. Han A. W. Johnson A. J. Holmes S. P. (2016). DADA2: High-resolution sample inference from Illumina amplicon data. Nat. Methods. 13, 581–583. doi: 10.1038/nmeth.3869 27214047 PMC4927377

[B3] Chen W. Chen X. Zhang Y. Wu H. Zhao D. (2024). Variation on gut microbiota diversity of endangered red pandas (*Ailurus fulgens*) living in captivity acrosss geographical latitudes. Front. Microbiol. 15:1420305. doi: 10.3389/fmicb.2024.1420305 39165571 PMC11333448

[B4] Danlin W. Qingxue G. Xiaorong W. Chunping L. Yuanbin Z. (2017). Effects of different altitudes on the nutrient and amino acid contents of bamboo (*fargesia denudat*a), staple food of the giant panda, in minshan, sichuan, china. Acta. Ecolo. Sinica. 37:stxb201607221496. doi: 10.5846/stxb201607221496

[B5] Delsuc F. Metcalf J. L. Parfrey L. W. Song S. J. Knight R. (2014). Convergence of gut microbiomes in myrmecophagous mammals. Mol. Ecol. 23, 1301–1317. doi: 10.1111/mec.12501 24118574

[B6] Deng F. Wang C. Li D. Peng Y. Deng L. Zhao Y. . (2023). The unique gut microbiome of giant pandas involved in protein metabolism contributes to the host's dietary adaption to bamboo. Microbiome 11:14. doi: 10.1186/s40168-023-01603-0 37580828 PMC10424351

[B7] Dhami B. Timilsina S. Adhikari A. Neupane B. Chhetri N. Sharma A. (2021). Research trends, conservation issues and approaches for the endangered red panda (*ailurus fulgens*): a systematic review of literatures across their home-range. J. Anim. Divers. 3, 57-68. doi: 10.52547/JAD.2021.3.2.6

[B8] Hall M. Beiko R. G. (2018). 16S rRNA gene analysis with QIIME2. Methods Mol. Biol. 1849, 113–129. doi: 10.1007/978-1-4939-8728-3_8 30298251

[B9] Han H. Wei W. Hu Y. Nie Y. G. Ji X. Yan L. . (2019). Diet evolution and habitat contraction of giant pandas via stable isotope analysis. Curr. biol. 29, 664-669. doi: 10.1016/j.cub.2018.12.051 30713107

[B10] Huang G. Shi W. Wang Q.u, l. Zuo Q. Y. Wang Z. Q. . (2023). Panda gut provides new insights into bacterial diversity, function, and resistome landscapes with implications for conservation. Microbiome 11:221. doi: 10.1186/s40168-023-01657-0 37805557 PMC10559513

[B11] Huang G. Wang L. Li J. Hou R. Wang M. Wang Z. . (2022). Seasonal shift of the gut microbiome synchronizes host peripheral circadian rhythm for physiological adaptation to a low-fat diet in the giant panda. Cell Rep. 38:110203. doi: 10.1016/j.celrep.2021.110203 35045306

[B12] Huang G. P. Wang X. Hu Y. B. Wu Q. Nie Y. G. Dong J. H. . (2021). Diet drives convergent evolution of gut microbiomes in bamboo-eating species. Sci. China. 64, 88–95. doi: 10.1007/s11427-020-1750-7 32617829

[B13] Huang X. L. Li H. B. Zhang L. Zhang X. Chen S. C. Yang Y. Y. . (2024). Comparative analysis of gut microbiota between wild and captive Guizhou Snub-Nosed Monkey (*Rhinopithecus brelichi*). *Ecol. Evol*. 14:e70690. doi: 10.1002/ece3.70690 PMC11631709 39664719

[B14] Jia J. Q. Hu S. L. Sun X. Cao Y. (2007). Lignin and cellulose contents of two typical thick-growing woody bamboos in sichuan. Acta. Botanica Boreali-Occidentalia Sinica. 27, 197–200. doi: 10.7606/j.issn.1000-4025.2007.01.0197

[B15] Kong F. L. Zhao J. C. Han H. H. Zeng B. Yang J. D. Si X. H. . (2014). Characterization of the gut microbiota in the Red Panda (*Ailurus fulgens*). *PLoS ONE* 9:e87885. doi: 10.1371/journal.pone.0087885 PMC3912123 24498390

[B16] Lei J. Huang Y. Yang S. Wu D. Zou L. (2021). Diet, habitat environment and lifestyle conversion affect the gut microbiomes of giant pandas. Sci. Total Environ. 770:145316. doi: 10.1016/j.scitotenv.2021.145316 33517011

[B17] Li Y. Guo W. Han S. Kong F. Wang C. Li D. . (2015). The evolution of the gut microbiota in the giant and the red pandas. Sci. Rep. 5:10185. doi: 10.1038/srep10185 25985413 PMC4434948

[B18] Li Y. Swaisgood R. R. Wei W. Nie Y. Hu Y. Yang X. . (2017). Withered on the stem: is bamboo a seasonally limiting resource for giant pandas? Environ. Sci. Pollut. R. 24, 1–10. doi: 10.1007/s11356-017-8746-6 28281076

[B19] Li Y. H. Yan Y. J. Fu H. G. Jin S. Y. He S. J. Wang Z. . (2023). Does diet or macronutrients intake drive the structure and function of gut microbiota? Front. Microbiol. 14:1126189. doi: 10.3389/fmicb.2023.1126189 36860485 PMC9970161

[B20] LiuL. LiangL. LiangH. WangM. ZhouW. MaiG. . (2025). Microbiome-metabolome generated bile acids gatekeep infliximab efficacy in Crohn's disease by licensing M1 suppression and Treg dominance. J. Adv. Res. 12, S2090-1232(25)00606-X. doi: 10.1016/j.jare.2025.08.01740812589

[B21] Liu T. T. Lu C. X. Yuan C. Y. Chen Y. Wu H. (2022). Screening, genetic modification of cellulose degrading strain from feces of red panda and optimization of polyhydroxybutyrate biosynthesis process. J. Tianjin Norm. Univ. (Nat. Sci. Ed.). 42, 45–50.

[B22] Liu X. Zhang M. S. Liu Z. S. (2011). Ecological research status of *Ailurus flugens*, China. Chin. J. Wildlife 32, 38–40. doi: 10.19711/j.cnki.issn2310-1490.2011.01.013

[B23] Lu Y. Zhang L. Liu X. Lan Y. Wu L. Wang J. . (2024). Red pandas with different diets and environments exhibit different gut microbial functional composition and capacity. Integr. Zool. 19:12813. doi: 10.1111/1749-4877.12813 38420673

[B24] Lyu F. Y. Han F. R. Ge C. L. Mao W. K. Chen L. Hu H. P. . (2023). OmicStudio: a composable bioinformatics cloud platform with real-time feedback that can generate high-quality graphs for publication. IMeta. 2:e85. doi: 10.1002/imt2.85 38868333 PMC10989813

[B25] Mckenney E. A. Maslanka M. Rodrigo A. Yoder A. D. (2017). Bamboo specialists from two mammalian orders (*primates, carnivora*) share a high number of low-abundance gut microbes. Microb. Ecol. 76, 1–13. doi: 10.1007/s00248-017-1114-8 29188302

[B26] Moeller A. H. Peeters M. Ndjango J. B. Li Y. Hahn B. H. Ochman H. (2013). Sympatric chimpanzees and gorillas harbor convergent gut microbial communities. Gen. Res. 23, 1715–1720. doi: 10.1101/gr.154773.113 23804402 PMC3787267

[B27] Nie Y. G. Wei F. W. Zhou W. L. Hu Y. B. Alistair M. (2019). Giant pandas are macronutritional carnivores. Curr. Biol. 29, 1677–1682. doi: 10.1016/j.cub.2019.03.067 31056385

[B28] Ning R. Li C. Xia M. Zhang Y. Gan Y. Huang Y. . (2024). Pseudomonas associated bacteria play a key role in obtaining nutrition from bamboo for the giant panda (*Ailuropoda melanoleuca*). *Microbiol. Spectr*. 12:23. doi: 10.1128/spectrum.03819-23 PMC10913395 38305171

[B29] Rajbhandari R. M. Forcina G. Manandhar P. Rajbhandari P. G. Napit R. Raut R. (2025). Gut microbiota diversity among humans, elephants, livestock and wild herbivores in chitwan national park bears implications for conservation medicine. Sci. Rep. 15:11596. doi: 10.1038/s41598-025-89402-5 40185849 PMC11971256

[B30] Ray S. S. Ali N. (2017). Biotic contamination and possible ways of sterilization: a review with reference to bamboo micropropagation. Braz. Arch. Biol. Techn. 60:e160485. doi: 10.1590/1678-4324-2016160485

[B31] Sayak M. Kristin E. Weimer S. Ray W. C. Jayaprakash C. Vieland V. J. . (2015). Host-to-host variation of ecological interactions in polymicrobial infections. Phys. Biol. 12:016003. doi: 10.1088/1478-3975/12/1/016003 25473880 PMC4269105

[B32] Shamjana U. Vasu D. A. Hembrom P. S. Nayak K. Grace T. (2024). The role of insect gut microbiota in host fitness, detoxification and nutrient supplementation. Anton. Leeuw. 117:71. doi: 10.1007/s10482-024-01970-0 38668783

[B33] Shan L. Wu Q. Wang L. Zhang L. Wei F. (2018). Lineage-specific evolution of bitter taste receptor genes in the giant and red pandas implies dietary adaptation. Integr. Zool. 13:12291. doi: 10.1111/1749-4877.12291 29168616 PMC5873442

[B34] Shen Y. Dinh H. V. Cruz E. R. Chen Z. Bartman C. R. Xiao T. . (2024). Mitochondrial atp generation is more proteome efficient than glycolysis. Nat. Chem. Biol. 20:31. doi: 10.1038/s41589-024-01571-y 38448734 PMC11925356

[B35] Wang H. Zhang Z. Hou R. Ayala J. Liu G. . (2017). A diet diverse in bamboo parts is important for giant panda (*Ailuropoda melanoleuca*) metabolism and health. Sci. Rep.. 7:3377. doi: 10.1038/s41598-017-03216-8 28611401 PMC5469786

[B36] Wang L. Yuan S. Nie Y. Zhao J. Wei F. (2020). Dietary flavonoids and the altitudinal preference of wild giant pandas in foping national nature reserve, china. Glob. Ecol. Conserv. 22:e00981. doi: 10.1016/j.gecco.2020.e00981

[B37] Wang Q. Garrity G. M. Tiedje J. M. Cole J. R. (2007). Naive Bayesian classifier for rapid assignment of rRNA sequences into the new bacterial taxonomy. Appl. Environ. Microbiol. 73, 5261–5267. doi: 10.1128/AEM.00062-07 17586664 PMC1950982

[B38] Wei F. Wang X. Wu Q. (2015). The giant panda gut microbiome. Trends Microbiol. 23, 450–452. doi: 10.1016/j.tim.2015.06.004 26143242

[B39] Wei F. W. Feng Z. J Wang Z. W. Zhou A. Hu J. C. (1999). Use of the nutrients in bamboo by the red panda (*Ailurus fulgens*). *J. Zool*.. 248, 535–541. doi: 10.1017/S0952836999008134

[B40] Wei G. Sudhanshu M. Jiangchao Z. Jingsi T. Bo Z. Fan K. (2018). Metagenomic study suggests that the gut microbiota of the giant panda (*Ailuropoda melanoleuca*) may not be specialized for fiber fermentation. Front. Microbiol. 9:229. doi: 10.3389/fmicb.2018.00229 29503636 PMC5820910

[B41] Williams C. L. Sparks D. L. Kouba A. J. Willard S. T. Dill-Mcfarland K. (2014). Giant and Red Pandas Utilize Distinct Microbial Communities for their Bamboo Diet Degradation. The 248th American Chemical Society. doi: 10.3969/j.issn.1009-6469.2014.01.018

[B42] Wu Y. Yao Y. Shen Y. Bai H. Zhang L. Zhang C. (2025). Nanoplastics Chronic Toxicity in Mice: disturbing the Homeostasis of Tryptophan Metabolism in Gut-Lung-Microbiota Axis. Small 21:e2412286. doi: 10.1002/smll.202412286 40351096

[B43] Xia W. C. Liu G. Q. Wang D. L. Chen H. Zhu L. F. Li D. Y. (2022). Functional convergence of yunnan snub-nosed monkey and bamboo-eating panda gut microbiomes revealing the driving by dietary flexibility on mammal gut microbiome. Comput. Struct. Biotec. 20, 685–699. doi: 10.1016/j.csbj.2022.01.011 35140888 PMC8814018

[B44] Xue Z. Zhang W. Wang L. Hou R. Zhang M. Fei L. . (2015). The bamboo-eating giant panda harbors a carnivore-like gut microbiota, with excessive seasonal variations. Mbio 6, e00022–e00015. doi: 10.1128/mBio.00022-15 25991678 PMC4442137

[B45] Yan Z. Wang H. Wang L. Liu X. Chen X. Liu D. . (2024). Functional responses of giant panda gut microbiota to high-fiber diets. Ursus 35:e5. doi: 10.2192/URSU-D-22-00017

[B46] Yao R. Yang Z. Zhang Z. Hu T. Zhu L. (2019). Are the gut microbial systems of giant pandas unstable? Heliyon 5:e02480. doi: 10.1016/j.heliyon.2019.e02480 31687574 PMC6819816

[B47] Zhang M. Zhou Y. Cui X. Zhu L. (2024). The potential of co-evolution and interactions of gut bacteria-phages in bamboo-eating pandas: insights from dietary preference-based metagenomic analysis. Microorganisms 12:23. doi: 10.3390/microorganisms12040713 38674657 PMC11051890

[B48] Zhang W. Liu W. Hou R. Zhang L. Schmitz-Esser S. Sun H. . (2018). Age-associated microbiome shows the giant panda lives on hemicelluloses, not on cellulose. ISME J. 12, 1319–1328. doi: 10.1038/s41396-018-0051-y 29391488 PMC5931968

[B49] Zhao X. Zhang Z. J. Wang L. Zhang Q. Kang L. W. Wang J. . (2024). Progress in research on the gut microflora of the red panda (*Ailurus fulgens*). *Microorganisms* 12:12030478. doi: 10.3390/microorganisms12030478 PMC10974310 38543529

[B50] Zheng J. Zhou X. Chen M. Liu Y. Li J. (2014). Introduction of an improved fecal bacterial genome DNA extracting test method. Anhui Med. Pharm. J. 1, 52–55.

[B51] Zhu L. Wu Q. Dai J. Zhang S. Wei F. (2011). Evidence of cellulose metabolism by the giant panda gut microbiome. PNAS 108, 17714–17719. doi: 10.1073/pnas.1017956108 22006317 PMC3203778

[B52] Zhu L. Yang Z. Yao R. Xu L. Chen H. Gu X. . (2018). Potential mechanism of detoxification of cyanide compounds by gut microbiomes of bamboo-eating pandas. MSPHERE 3:e00229–e00218. doi: 10.1128/mSphere.00229-18 29898983 PMC6001608

[B53] Zuñiga C. Levering J. Antoniewicz M. R. Guarnieri M. T. Betenbaugh M. J. Zengler K. (2017). Predicting dynamic metabolic demands in the photosynthetic eukaryote Chlorella vulgaris. Plant Physiol. 176, 450–462. doi: 10.1104/pp.17.00605 28951490 PMC5761767

