# Supplementary material for: Divergent gut microbial metabolism supports niche partitioning in giant and red pandas
Source: Front Microbiol. 2025 Nov 25;16:1698108. doi: 10.3389/fmicb.2025.1698108 (PMC12687339; doi:10.3389/fmicb.2025.1698108)
Supplement: Supplementary file 1 [file Supplementary_file_1.docx]

Supplementary Material

Table S1. The sample information

| Group | Sample Name | Sex | Age | Sampling date |
| --- | --- | --- | --- | --- |
| RP | B3 | M | 5 | 2/14/2024 |
|  | B13 | F | 6 | 2/14/2024 |
|  | B14 | F | 7 | 2/14/2024 |
|  | B20 | M | 5 | 2/14/2024 |
|  | B23 | M | 8 | 2/14/2024 |
|  | B24 | F | 9 | 2/14/2024 |
| GP | P5 | F | 11 | 2/14/2024 |
|  | P9 | F | 14 | 2/14/2024 |
|  | P11 | M | 13 | 2/14/2024 |
|  | P16 | M | 10 | 2/14/2024 |
|  | P17 | F | 13 | 2/14/2024 |

Table S2. The sequencing information

| Group | Sample Name | Reads after QC （before rarefaction） | Reads after rarefaction | Base(nt) | AvgLen(bp) |
| --- | --- | --- | --- | --- | --- |
| RP | B3 | 12620 | 11000 | 18482721 | 1464 |
|  | B13 | 11124 | 11000 | 16176078 | 1454 |
|  | B14 | 12425 | 11000 | 18157665 | 1461 |
|  | B20 | 11215 | 11000 | 16436090 | 1465 |
|  | B23 | 12727 | 11000 | 18372607 | 1443 |
|  | B24 | 12066 | 11000 | 17465877 | 1447 |
| GP | P5 | 12833 | 11000 | 18887742 | 1471 |
|  | P9 | 11147 | 11000 | 16401373 | 1471 |
|  | P11 | 11250 | 11000 | 16485718 | 1465 |
|  | P16 | 12501 | 11000 | 18327104 | 1466 |
|  | P17 | 13430 | 11000 | 19726144 | 1468 |

Table S3. The difference of gut bacterial Alpha diversity between GP and RP group

| Alpha Index | GP | | RP | | p.adj |
| --- | --- | --- | --- | --- | --- |
|  | Mean | sd | Mean | sd |  |
| shannon | 7.185 | 0.528 | 4.258 | 0.771 | 0.0043 |
| simpson | 0.969 | 0.023 | 0.784 | 0.106 | 0.0043 |
| chao1 | 1060.580 | 140.433 | 1263.776 | 407.743 | 0.6623 |
| ace | 864.676 | 110.182 | 970.920 | 311.421 | 0.7922 |

Table S4. The difference gut phyla and genera between GP and RP group

| Phyla | Group | LDA score (log10) | *p* |
| --- | --- | --- | --- |
| Firmicutes | RP | 5.451 | 0.006 |
| Proteobacteria | GP | 5.262 | 0.006 |
| Planctomycetes | GP | 4.401 | 0.006 |
| Actinobacteria | GP | 4.401 | 0.018 |
| Acidobacteriota | GP | 4.240 | 0.006 |
| Bacteroidota | GP | 4.117 | 0.018 |
| Genera |  |  |  |
| Streptococcus | RP | 5.280 | 0.006 |
| Gemella | RP | 4.042 | 0.006 |
| Sphingomonas | GP | 4.732 | 0.006 |
| Methylobacterium_Methylorubrum | GP | 4.404 | 0.006 |
| Tundrisphaera | GP | 4.351 | 0.006 |
| Terriglobus | GP | 4.055 | 0.006 |

Table S5. The difference of KEGG level2 functional metabolism between GP and RP groups

| KEGG level2 | Mean(GP) | sd(GP) | Mean(RP) | sd(RP) | *p*.adj |
| --- | --- | --- | --- | --- | --- |
| Cellular Processes; Cell Growth and Death | 0.70 | 0.07 | 0.58 | 0.05 | 0.011 |
| Cellular Processes; Cell Motility | 2.95 | 0.35 | 1.57 | 0.60 | 0.006 |
| Cellular Processes; Transport and Catabolism | 0.32 | 0.01 | 0.23 | 0.04 | 0.006 |
| Environmental Information Processing; Membrane Transport | 10.66 | 0.59 | 13.87 | 0.49 | 0.006 |
| Environmental Information Processing; Signal Transduction | 2.35 | 0.13 | 1.71 | 0.13 | 0.006 |
| Environmental Information Processing; Signaling Molecules and Interaction | 0.19 | 0.01 | 0.23 | 0.03 | 0.020 |
| Genetic Information Processing; Replication and Repair | 6.87 | 0.08 | 8.20 | 0.46 | 0.006 |
| Genetic Information Processing; Transcription | 2.12 | 0.08 | 2.52 | 0.23 | 0.011 |
| Genetic Information Processing; Translation | 4.08 | 0.11 | 5.29 | 0.34 | 0.006 |
| Metabolism; Amino Acid Metabolism | 10.25 | 0.36 | 9.05 | 0.39 | 0.006 |
| Metabolism; Biosynthesis of Other Secondary Metabolites | 1.04 | 0.01 | 0.92 | 0.04 | 0.006 |
| Metabolism; Lipid Metabolism | 3.65 | 0.09 | 3.01 | 0.16 | 0.006 |
| Metabolism; Metabolism of Other Amino Acids | 1.98 | 0.04 | 1.69 | 0.07 | 0.006 |
| Metabolism; Metabolism of Terpenoids and Polyketides | 2.22 | 0.07 | 1.89 | 0.11 | 0.006 |
| Metabolism; Nucleotide Metabolism | 3.11 | 0.03 | 3.88 | 0.27 | 0.006 |
| Metabolism; Xenobiotics Biodegradation and Metabolism | 3.74 | 0.46 | 2.57 | 0.34 | 0.006 |
| Metabolism; Carbohydrate Metabolism | 0.10 | 0.00 | 0.11 | 0.01 | 0.016 |
| Organismal Systems; Circulatory System | 0.04 | 0.01 | 0.01 | 0.00 | 0.006 |
| Organismal Systems; Endocrine System | 0.40 | 0.05 | 0.27 | 0.03 | 0.006 |
| Organismal Systems; Excretory System | 0.04 | 0.00 | 0.02 | 0.01 | 0.006 |

Table S6. The spearman correlation between different genera and KEGG level2 functional metabolism in all gut samples

| Metabolic pathway | *Gemella* | *MethylobacteriumMethylorubrum* | *Sphingomonas* | *Streptococcus* | *Terriglobus* | *Tundrisphaera* |
| --- | --- | --- | --- | --- | --- | --- |
|  | Spearman *r* | | | | | |
| Cell Growth and Death | -0.798 | 0.882 | 0.709 | -0.682 | 0.826 | 0.800 |
| Cell Motility | -0.817 | 0.755 | 0.845 | -0.873 | 0.706 | 0.864 |
| Transport and Catabolism | -0.798 | 0.827 | 0.718 | -0.655 | 0.835 | 0.700 |
| Membrane Transport | 0.725 | -0.709 | -0.700 | 0.836 | -0.743 | -0.818 |
| Signal Transduction | -0.936 | 0.918 | 0.818 | -0.764 | 0.752 | 0.927 |
| Signaling Molecules and Interaction | 0.807 | -0.882 | -0.509 | 0.600 | -0.541 | -0.673 |
| Replication and Repair | 0.899 | -0.882 | -0.800 | 0.727 | -0.716 | -0.827 |
| Transcription | 0.798 | -0.864 | -0.600 | 0.627 | -0.688 | -0.709 |
| Translation | 0.835 | -0.800 | -0.736 | 0.818 | -0.624 | -0.791 |
| Amino Acid Metabolism | -0.853 | 0.773 | 0.791 | -0.745 | 0.651 | 0.727 |
| Biosynthesis of Other Secondary Metabolites | -0.789 | 0.773 | 0.736 | -0.709 | 0.743 | 0.864 |
| Lipid Metabolism | -0.807 | 0.773 | 0.855 | -0.673 | 0.844 | 0.773 |
| Metabolism of Other Amino Acids | -0.835 | 0.773 | 0.755 | -0.791 | 0.651 | 0.736 |
| Metabolism of Terpenoids and Polyketides | -0.908 | 0.864 | 0.736 | -0.591 | 0.734 | 0.755 |
| Nucleotide Metabolism | 0.817 | -0.727 | -0.718 | 0.809 | -0.560 | -0.836 |
| Xenobiotics Biodegradation and Metabolism | -0.890 | 0.800 | 0.773 | -0.736 | 0.624 | 0.800 |
| Carbohydrate Metabolism | 0.569 | -0.545 | -0.527 | 0.709 | -0.477 | -0.709 |
| Circulatory System | -0.908 | 0.900 | 0.864 | -0.745 | 0.835 | 0.918 |
| Endocrine System | -0.817 | 0.800 | 0.682 | -0.718 | 0.697 | 0.773 |
| Excretory System | -0.679 | 0.655 | 0.709 | -0.800 | 0.725 | 0.773 |
|  | Spearman *p* | | | | | |
| Cell Growth and Death | 0.003 | 0.001 | 0.019 | 0.025 | 0.002 | 0.005 |
| Cell Motility | 0.002 | 0.010 | 0.002 | 0.001 | 0.015 | 0.001 |
| Transport and Catabolism | 0.003 | 0.003 | 0.017 | 0.034 | 0.001 | 0.021 |
| Membrane Transport | 0.012 | 0.019 | 0.021 | 0.003 | 0.009 | 0.004 |
| Signal Transduction | 0.000 | 0.000 | 0.004 | 0.009 | 0.008 | 0.000 |
| Signaling Molecules and Interaction | 0.003 | 0.001 | 0.114 | 0.056 | 0.085 | 0.028 |
| Replication and Repair | 0.000 | 0.001 | 0.005 | 0.015 | 0.013 | 0.003 |
| Transcription | 0.003 | 0.001 | 0.056 | 0.044 | 0.019 | 0.019 |
| Translation | 0.001 | 0.005 | 0.013 | 0.004 | 0.040 | 0.006 |
| Amino Acid Metabolism | 0.001 | 0.008 | 0.006 | 0.012 | 0.030 | 0.015 |
| Biosynthesis of Other Secondary Metabolites | 0.004 | 0.008 | 0.013 | 0.019 | 0.009 | 0.001 |
| Lipid Metabolism | 0.003 | 0.008 | 0.002 | 0.028 | 0.001 | 0.008 |
| Metabolism of Other Amino Acids | 0.001 | 0.008 | 0.010 | 0.006 | 0.030 | 0.013 |
| Metabolism of Terpenoids and Polyketides | 0.000 | 0.001 | 0.013 | 0.061 | 0.010 | 0.010 |
| Nucleotide Metabolism | 0.002 | 0.015 | 0.017 | 0.004 | 0.073 | 0.003 |
| Xenobiotics Biodegradation and Metabolism | 0.000 | 0.005 | 0.008 | 0.013 | 0.040 | 0.005 |
| Carbohydrate Metabolism | 0.068 | 0.087 | 0.100 | 0.019 | 0.138 | 0.019 |
| Circulatory System | 0.000 | 0.000 | 0.001 | 0.012 | 0.001 | 0.000 |
| Endocrine System | 0.002 | 0.005 | 0.025 | 0.017 | 0.017 | 0.008 |
| Excretory System | 0.022 | 0.034 | 0.019 | 0.005 | 0.012 | 0.008 |

Table S7. The Univariate Linear Regression correlation between different genera and KEGG level2 functional metabolism in all gut samples

| Metabolic pathway | | | *Gemella* | *MethylobacteriumMethylorubrum* | | | *Sphingomonas* | *Streptococcus* | | | *Terriglobus* | *Tundrisphaera* | |
| --- | --- | --- | --- | --- | --- | --- | --- | --- | --- | --- | --- | --- | --- |
|  |  |  | Univariate Linear Regression *R^2^* | | | | | | | | | | |
| Cell Growth and Death | | | 0.148 | 0.463 | | | 0.663 | 0.625 | | | 0.657 | 0.630 | |
| Cell Motility | | | 0.822 | 0.319 | | | 0.710 | 0.507 | | | 0.518 | 0.469 | |
| Transport and Catabolism | | | 0.093 | 0.186 | | | 0.580 | 0.512 | | | 0.228 | 0.206 | |
| Membrane Transport | | | 0.563 | 0.340 | | | 0.927 | 0.638 | | | 0.596 | 0.533 | |
| Signal Transduction | | | 0.422 | 0.543 | | | 0.902 | 0.737 | | | 0.610 | 0.550 | |
| Signaling Molecules and Interaction | | | 0.090 | 0.844 | | | 0.438 | 0.569 | | | 0.126 | 0.092 | |
| Replication and Repair | | | 0.416 | 0.702 | | | 0.751 | 0.711 | | | 0.347 | 0.287 | |
| Transcription | | | 0.078 | 0.444 | | | 0.531 | 0.565 | | | 0.204 | 0.167 | |
| Translation | | | 0.593 | 0.567 | | | 0.774 | 0.692 | | | 0.326 | 0.267 | |
| Amino Acid Metabolism | | | 0.455 | 0.524 | | | 0.624 | 0.573 | | | 0.161 | 0.128 | |
| Biosynthesis of Other Secondary Metabolites | | | 0.380 | 0.372 | | | 0.805 | 0.642 | | | 0.402 | 0.339 | |
| Lipid Metabolism | | | 0.343 | 0.361 | | | 0.811 | 0.632 | | | 0.337 | 0.298 | |
| Metabolism of Other Amino Acids | | | 0.617 | 0.401 | | | 0.773 | 0.627 | | | 0.300 | 0.257 | |
| Metabolism of Terpenoids and Polyketides | | | 0.216 | 0.628 | | | 0.672 | 0.667 | | | 0.197 | 0.155 | |
| Nucleotide Metabolism | | | 0.765 | 0.487 | | | 0.751 | 0.603 | | | 0.358 | 0.299 | |
| Xenobiotics Biodegradation and Metabolism | | | 0.385 | 0.475 | | | 0.581 | 0.537 | | | 0.098 | 0.069 | |
| Carbohydrate Metabolism | | | 0.509 | 0.243 | | | 0.375 | 0.262 | | | 0.337 | 0.299 | |
| Circulatory System | | | 0.421 | 0.464 | | | 0.923 | 0.709 | | | 0.555 | 0.507 | |
| Endocrine System | | | 0.398 | 0.402 | | | 0.600 | 0.575 | | | 0.113 | 0.075 | |
| Excretory System | | | 0.561 | 0.162 | | | 0.693 | 0.532 | | | 0.305 | 0.257 | |
|  | | | Univariate Linear Regression *p* | | | | | | | | | | |
| Cell Growth and Death | | | 0.243 | 0.021 | | | 0.002 | 0.004 | | | 0.003 | 0.004 | |
| Cell Motility | | | 0.000 | 0.070 | | | 0.001 | 0.014 | | | 0.013 | 0.020 | |
| Transport and Catabolism | | | 0.361 | 0.186 | | | 0.007 | 0.013 | | | 0.137 | 0.161 | |
| Membrane Transport | | | 0.008 | 0.060 | | | <0.0001 | 0.003 | | | 0.005 | 0.011 | |
| Signal Transduction | | | 0.031 | 0.010 | | | <0.0001 | 0.001 | | | 0.005 | 0.009 | |
| Signaling Molecules and Interaction | | | 0.371 | <0.0001 | | | 0.026 | 0.007 | | | 0.285 | 0.366 | |
| Replication and Repair | | | 0.032 | 0.001 | | | 0.001 | 0.001 | | | 0.057 | 0.089 | |
| Transcription | | | 0.406 | 0.025 | | | 0.011 | 0.008 | | | 0.163 | 0.212 | |
| Translation | | | 0.006 | 0.008 | | | <0.0001 | 0.002 | | | 0.067 | 0.104 | |
| Amino Acid Metabolism | | | 0.023 | 0.012 | | | 0.004 | 0.007 | | | 0.221 | 0.280 | |
| Biosynthesis of Other Secondary Metabolites | | | 0.043 | 0.046 | | | <0.0001 | 0.003 | | | 0.036 | 0.060 | |
| Lipid Metabolism | | | 0.058 | 0.051 | | | <0.0001 | 0.004 | | | 0.061 | 0.082 | |
| Metabolism of Other Amino Acids | | | 0.004 | 0.037 | | | <0.0001 | 0.004 | | | 0.081 | 0.112 | |
| Metabolism of Terpenoids and Polyketides | | | 0.150 | 0.004 | | | 0.002 | 0.002 | | | 0.172 | 0.231 | |
| Nucleotide Metabolism | | | <0.0001 | 0.017 | | | 0.001 | 0.005 | | | 0.052 | 0.082 | |
| Xenobiotics Biodegradation and Metabolism | | | 0.042 | 0.019 | | | 0.006 | 0.010 | | | 0.348 | 0.436 | |
| Carbohydrate Metabolism | | | 0.014 | 0.124 | | | 0.045 | 0.108 | | | 0.061 | 0.082 | |
| Circulatory System | | | 0.031 | 0.021 | | | <0.0001 | 0.001 | | | 0.009 | 0.014 | |
| Endocrine System | | | 0.037 | 0.036 | | | 0.005 | 0.007 | | | 0.312 | 0.414 | |
| Excretory System | | | 0.008 | 0.220 | | | 0.002 | 0.011 | | | 0.078 | 0.112 | |
|  | | | Univariate Linear Regression Adjusted *p* value by Benjamini-Hochberg | | | | | | | | | | |
| Cell Growth and Death | | 0.265 | | | 0.040 | 0.013 | | | 0.015 | 0.013 | | | 0.015 |
| Cell Motility | | 0.001 | | | 0.096 | 0.008 | | | 0.028 | 0.027 | | | 0.039 |
| Transport and Catabolism | | 0.377 | | | 0.212 | 0.020 | | | 0.028 | 0.165 | | | 0.190 |
| Membrane Transport | | 0.021 | | | 0.086 | 0.000 | | | 0.015 | 0.018 | | | 0.025 |
| Signal Transduction | | 0.053 | | | 0.024 | 0.000 | | | 0.006 | 0.016 | | | 0.023 |
| Signaling Molecules and Interaction | | 0.380 | | | 0.000 | 0.047 | | | 0.021 | 0.305 | | | 0.378 |
| Replication and Repair | | 0.055 | | | 0.009 | 0.006 | | | 0.008 | 0.084 | | | 0.114 |
| Transcription | 0.413 | | | | 0.046 | 0.025 | | | 0.021 | 0.190 | | | 0.240 |
| Translation | 0.018 | | | | 0.021 | 0.000 | | | 0.009 | 0.092 | | | 0.131 |
| Amino Acid Metabolism | 0.042 | | | | 0.026 | 0.015 | | | 0.020 | 0.246 | | | 0.303 |
| Biosynthesis of Other Secondary Metabolites | 0.069 | | | | 0.071 | 0.000 | | | 0.015 | 0.060 | | | 0.086 |
| Lipid Metabolism | 0.085 | | | | 0.077 | 0.000 | | | 0.015 | 0.086 | | | 0.106 |
| Metabolism of Other Amino Acids | 0.016 | | | | 0.060 | 0.000 | | | 0.015 | 0.106 | | | 0.137 |
| Metabolism of Terpenoids and Polyketides | 0.178 | | | | 0.015 | 0.012 | | | 0.012 | 0.198 | | | 0.255 |
| Nucleotide Metabolism | 0.000 | | | | 0.034 | 0.006 | | | 0.017 | 0.078 | | | 0.106 |
| Xenobiotics Biodegradation and Metabolism | 0.067 | | | | 0.037 | 0.020 | | | 0.025 | 0.367 | | | 0.436 |
| Carbohydrate Metabolism | 0.028 | | | | 0.150 | 0.071 | | | 0.134 | 0.086 | | | 0.106 |
| Circulatory System | 0.053 | | | | 0.040 | 0.000 | | | 0.008 | 0.022 | | | 0.028 |
| Endocrine System | 0.061 | | | | 0.060 | 0.017 | | | 0.020 | 0.331 | | | 0.417 |
| Excretory System | 0.021 | | | | 0.246 | 0.009 | | | 0.025 | 0.106 | | | 0.137 |


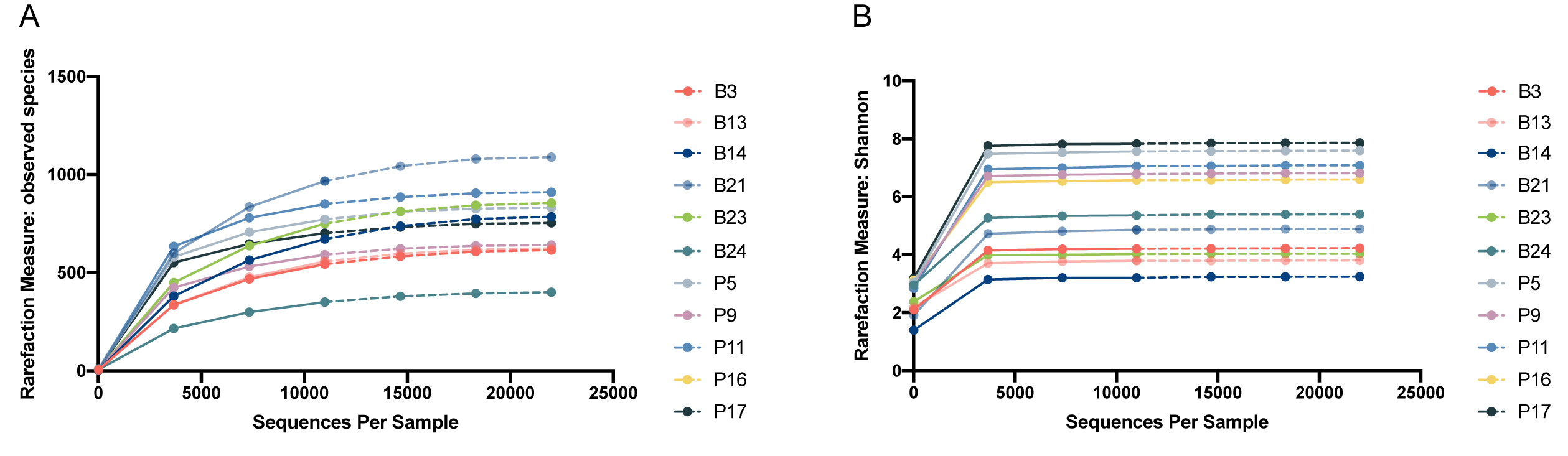


Figure S1. Rarefaction curves evaluating the adequacy of sequencing depth. A. Rarefaction curves based on the number of observed amplicon sequence variants (ASVs), representing species richness. B. Rarefaction curves based on the Shannon diversity index, which reflects both species richness and evenness. Each curve represents an individual sample. The solid segment of each curve is based on actual subsampled data, while the dashed segment represents extrapolation beyond the obtained sequencing depth. The rarefaction depth of 11,000 reads per sample used in this study. The plateauing trends of both the observed ASVs and Shannon curves at this depth confirm that the sequencing effort was sufficient to capture the majority of microbial diversity within the samples.


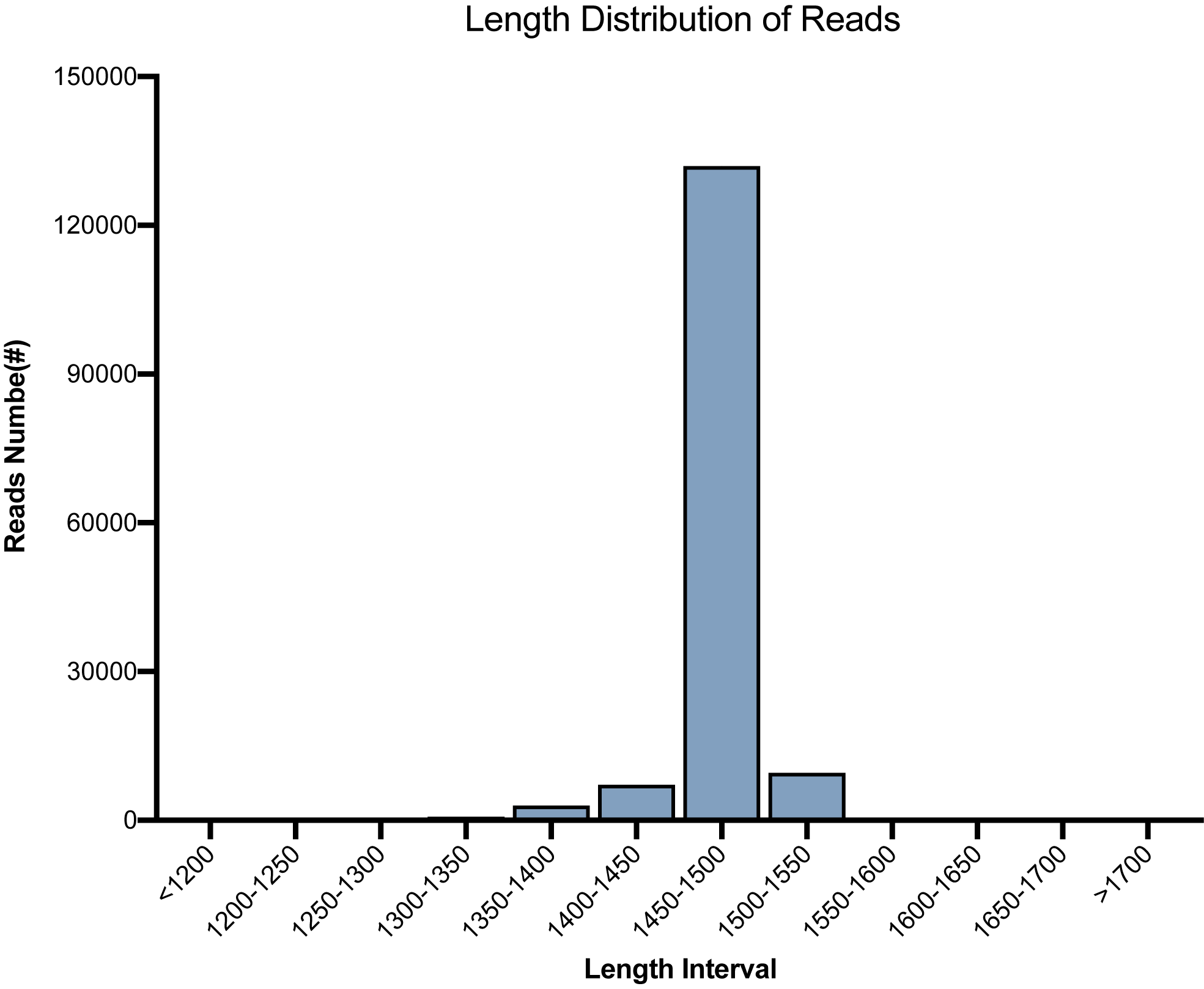


Figure S2. The length distribution of reads from all 11 samples.
